# The Need for Creating a Unified Knowledge of Cardiovascular Diseases in Latin America

**DOI:** 10.21470/1678-9741-2022-0954

**Published:** 2022

**Authors:** Manuel Urina-Jassir, Maria Alejandra Jaimes-Reyes, Samuel Martinez-Vernaza, Miguel Urina-Triana

**Affiliations:** 1 Department of Clinical Research, Fundación del Caribe para la Investigación Biomédica, Barranquilla, Colombia.; 2 Infectious Disease Unit, Hospital Universitario San Ignacio, Bogotá D.C., Colombia.; 3 Faculty of Health Sciences, Universidad Simón Bolívar, Barranquilla, Colombia.

Cardiovascular diseases (CVDs) have persistently been the principal cause of disease burden and mortality throughout the world as well as in Latin America (LATAM)^[1,2]^. Congruently, as CVDs continue to grow, the research production in this discipline has followed the same trend; global CVD publications have been increasing in the last decades^[3,4]^. However, as with other health-related topics, disparities in the quantity of research exist when comparing low- and middle-income countries with high-income nations^[3,4]^.

Despite the simultaneous increasing trend in CVD research output in LATAM, this region is clearly behind in terms of publications when compared to North America or Europe. A bibliometric analysis of CVD papers in PubMed® identified that 4% of them were from LATAM, as opposed to 40% from European countries^[5]^. Even between Latin American countries, disparities also exist, with Argentina, Brazil, and Mexico being the most represented countries in published research in the region^[5,6]^.

As a reader, when assessing collaborative or pooled studies such as clinical trials or systematic reviews, there seems to be a lower representation of the Latin American population, publications, and/or journals. This could possibly be explained due to language barriers, as Spanish or Portuguese publications are excluded in many cases from systematic reviews, or due to the lack of indexation of many Latin American medical journals in major international databases such as PubMed®/MEDLINE®^[7,8]^.

In our recent experience, we identified that information about the Latin American population’s characteristics of infective endocarditis (IE) was lacking. Therefore, our approach was to conduct a systematic review including the two major international databases, as well as two regional Latin American databases. We identified and described compiled information on the major characteristics of IE that hopefully will be useful, or at least be a starting point for clinicians, researchers, and local guidelines^[9]^. Efforts to develop a unified knowledge, via systematic reviews, have also been conducted by other authors in the field of CVDs in LATAM^[10,11]^. For instance, Ciapponi et al.^[10]^ assessed the burden of heart failure (HF) in the region. They described crucial data on the prevalence, readmission, and mortality rates due to HF providing an overall assessment of this disease situation in LATAM. Similarly, Carrillo-Larco et al.^[11]^ produced essential information on the prevalence of dyslipidemia as well as the trends among the lipid profile levels in Latin American population-based studies. Besides the mentioned value of these systematic reviews, this type of study is also key to identify what is missing in the literature, such as more robust multicenter studies, registries, and/or population studies^[9,10]^.

Population-based or national registries in CVDs have proven to be useful in the generation of information and research that will ultimately improve the quality of patient care^[12,13]^. On the topic of IE, a great example of multicenter studies is the “Infective Endocarditis in Argentina” study, which has now published its third cohort (EIRA-3), in which 48 centers throughout that nation reported information on IE to generate updated information on this disease^[14]^. Likewise in the HF literature, based on the need for this type of work, national registries such as those in Argentina^[15]^, Brazil^[16]^, and Colombia^[17]^ have been developed. Moreover, the “Brazilian Registry of Adults Undergoing Cardiovascular Surgery” is an example in which the clinical outcomes of cardiovascular interventions can be systematically assessed^[18]^. In addition to these examples of national efforts, collaborations between researchers from distinct Latin American countries (with or without researchers from other regions) have also been generated or are being conducted on topics such as traditional risk factors and CVD (PURE study^[19]^), coronary heart disease (ACCESS^[20]^ and INTERASPIRE^[21]^ registries), and, more recently, coronavirus disease 2019 (COVID-19) cardiovascular complications (CARDIO-COVID 19-20 registry^[22]^). These examples demonstrate that research collaborations between Latin American researchers are feasible and should continue to grow over time.

There are multiple benefits of developing local and regional research such as obtaining truthful information about CVDs as well as identifying the needs in healthcare or research^[6]^, but most importantly, there has been described an inverse relationship between the number of publications and cardiovascular burden^[4]^. In other words, research output may be related to an improvement in CVD-associated death rates and disability-adjusted life years^[4]^, which should be the most important motivator for researchers.

In conclusion, as a region, LATAM is heading in the right direction in CVD research, but there is still work to do. The current momentum and research efforts should be continued with the aim of improving and continuously creating consolidated research in the region. This could be attained by using systematic reviews and meta-analyses, but more importantly, by developing collaborative strategies between researchers to produce high-quality primary research data.
